# Evaluation of the accuracy of fully guided implant placement by undergraduate students and postgraduate dentists: a comparative prospective clinical study

**DOI:** 10.1186/s40729-024-00526-1

**Published:** 2024-02-07

**Authors:** Ece Atay, Jeremias Hey, Florian Beuer, Mats Wernfried Heinrich Böse, Ramona Schweyen

**Affiliations:** 1 Department of Prosthodontics, Geriatric Dentistry and Craniomandibular Disorders, Charité-Universitätsmedizin Berlin, Corporate Member of Freie Universität Berlin and Humboldt-Universität Zu Berlin, Aßmannshauser Str. 4-6, 14197 Berlin, Germany; 2 Department of Prosthetic Dentistry, University School of Dental Medicine, Martin Luther University Halle-Wittenberg, Magdeburger Str. 16, 06112 Halle, Germany; 3 Mund. Kiefer. Gesicht. Bremen, Gröpelinger Heerstr. 406, 28239 Bremen, Germany

**Keywords:** Guided implant placement, Undergraduate students, Dental education, Implant accuracy

## Abstract

**Purpose:**

This study aimed to assess the accuracy of implant placement through three-dimensional planning and fully guided insertion, comparing outcomes between undergraduate and postgraduate surgeons.

**Methods:**

Thirty-eight patients requiring 42 implants in posterior single-tooth gaps were enrolled from the University Clinic for Prosthodontics at the Martin Luther University Halle Wittenberg and the Department of Prosthodontics, Geriatric Dentistry, and Craniomandibular Disorders of Charité University Medicine, Berlin. Twenty-two implants were placed by undergraduate students (*n* = 18), while 20 implants were placed by trainee postgraduate dentists (*n* = 5). Pre-operative intraoral scans and cone beam computed tomography images were performed for implant planning and surgical template fabrication. Postoperative intraoral scans were superimposed onto the original scans to analyze implant accuracy in terms of apical, coronal, and angular deviations, as well as vertical discrepancies.

**Results:**

In the student group, two implant insertions were performed by the assistant dentist because of intraoperative complications and, thus, were excluded from further analysis. For the remaining implants, no statistically significant differences were observed between the dentist and student groups in terms of apical (*p* = 0.245), coronal (*p* = 0.745), or angular (*p* = 0.185) implant deviations, as well as vertical discrepancies (*p* = 0.433).

**Conclusions:**

This study confirms the viability of fully guided implant placement by undergraduate students, with comparable accuracy to postgraduate dentists. Integration into dental education can prepare students for implant procedures, expanding access and potentially reducing costs in clinical practice. Collaboration is essential for safe implementation, and future research should explore long-term outcomes and patient perspectives, contributing to the advancement of dental education and practice.

*Trial registration:* DRKS, DRKS00023024, Registered 8 September 2020—Retrospectively registered, https://drks.de/search/de/trial/DRKS00023024.

## Background

Implants have been established as standard therapeutic tools in restorative dentistry [1]. Successful implant placement, defined as "ideal clinical conditions over a period of at least 12 months for implants serving as prosthetic abutments" [2], demand specific pre-requisites, such as healthy mucosal and bone tissue conditions in the area of the future implant site, presurgical evaluation of bone density, accurate measurement of the bone volume to determine the ideal implant dimensions, selection of the correct implant type with adequate surface treatment, experienced execution of the implant placement to achieve sufficient primary stability, suitable soft-tissue management during and after exposure, an appropriate design of the prosthetic restoration, and the patient's motivation to carry out the required oral hygiene and follow-up care [1, 3–5]. In addition to the objective parameters, the patient's subjective perception of the treatment, referred to as the patient reported outcome, and the change in oral health-related quality of life are becoming increasingly important. Various studies have demonstrated an improvement in the subjective perception of oral health-related quality of life as a result of the fabrication of implant-supported dental prostheses [6–8].

Traditionally, complex surgery, such as implant planning and placement were limited to experienced oral surgeons due to the difficulties of maintaining accuracy and safe distances from critical anatomical structures [1, 9]. Advancements in cone beam computed tomography (CBCT) and scan-guided three-dimensional implant planning have enabled accurate positioning, offering predictability even for less experienced practitioners and is particularly recommended in complex cases [10–12].

Recent studies favor three-dimensional implant planning and full-guided implant preparation, demonstrating superior accuracy compared to freehand or pilot-drill-guided insertion, benefiting both experienced and inexperienced practitioners [11, 13, 14]. Recent surveys have highlighted that the fundamentals of implant placement, including simple procedures, should be integrated into contemporary undergraduate dental education and have been part of the program at some universities for several years [14–17]. Students often learn the basics of implant planning, and at some universities, they also learn independent implant placement [18, 19].

Three-dimensional treatment planning with the fabrication of a surgical guide is recommended as a method for implementing the requirements for an ideal implant position [20–22]. Therefore, this protocol is considered ideal, particularly for beginners. However, this method also has various sources of error, which can result in an incorrect implant position despite all precautionary measures [21]. Therefore, even experienced surgeons may encounter deviations in accuracy between the planned and actual implant positions. These discrepancies can be influenced by the dental situation, fixation of the surgical guide, the characteristics of the jaw to be implanted, and the length and design of the implant [23, 24]. Additionally, various complications have been described in the literature, mostly due to limited visibility and accessibility to the surgical site, incorrect fit of the surgical guide, or the flapless approach [25, 26]. Particularly for beginners, the lack of surgical experience may lead to delayed or no recognition of intraoperative complications with a reduced overview [9].

Whether undergraduate students differ significantly from surgically inexperienced beginners in terms of qualifications remains questionable. For the latter, a range of different postgraduate training opportunities are offered, but to obtain the degree, these usually require the novice to place the implant initially under supervision and then deal solely with the associated risks alone. This raises the question of whether students should already gain experience with three-dimensional planning and fully guided preparation of the implant cavity during their undergraduate programs to become familiar with possible difficulties and thus learn solutions from their teachers. Whether undergraduate students are competent to plan or insert implants independently remains questionable.

This study aimed to compare the accuracy of implant placement through three-dimensional planning and fully guided insertion by undergraduate students and postgraduate dentists. Undergraduate students were responsible for planning and producing the CBCT-supported drilling guide, and they performed the fully guided implantation under the supervision of an experienced dentist. Postgraduate dentists planned and performed the fully guided implantation independently. Our hypothesis posited that there would be no significant difference in implant accuracy between the two groups. The primary outcome focused on evaluating implant accuracy depending on the operator. Additionally, secondary outcomes analyzed the influence of additional parameters, such as gap situation, assignment to the jaw, and implant length.

## Methods

**Fig. 1:**
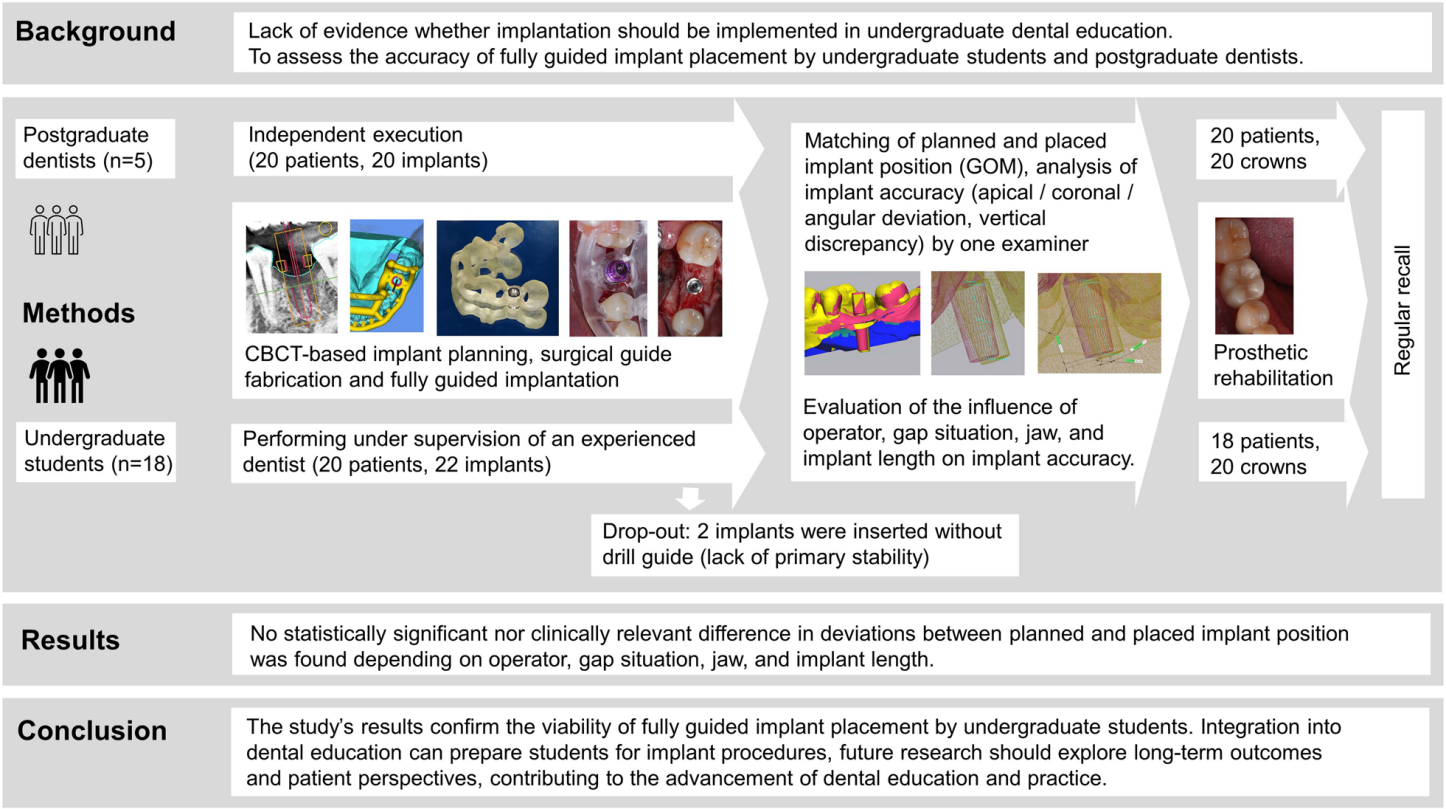
Schematic of the study protocol

The study was performed according to the requirements of the Declaration of Helsinki and approved by the ethics committees of Martin Luther University Halle-Wittenberg and the University Medicine Berlin Charité (No. 2020-041 and EA4/111/19). The study protocol is illustrated in Fig. 1.

### Patient recruitment and operators

Patients with an indication for an implant in a posterior single-tooth gap were enrolled from the University Clinic for Prosthodontics of Martin Luther University Halle Wittenberg and the Prosthodontics, Geriatric Dentistry and Craniomandibular Disorders of Charité University Medicine, Berlin between January 2018 and 2022. To participate in the study, the following inclusion criteria had to be met: (1) at least 18 years of age; (2) American Society of Anesthesiologists classification 1 or 2; (3) tooth extraction at least 3 months before ( delayed placement); and (4) an adequate amount of bone volume to place the implant without bone augmentation (2 mm bone circumferentially around the implant according to preoperative planning) [27–29]. Patients with major systemic diseases, untreated or uncontrolled caries, periodontal disease, a need for augmentation (bone grafting or sinus lift) in the planned implant area, current pregnancy, or noncompliance were excluded. All the patients provided written informed consent to participate in this study.

In half of the cases, implant planning, fully guided template production, and implant insertion were performed by postgraduate dentists in specialty training with more than two years of clinical practice. All postgraduate dentists finished a modular postgraduate training course in dental implantology and had already restored and placed more than 10 implants until participating in this study. In the other half of the cases, undergraduate students performed implant planning, fully guided template production, and implant insertion under the supervision of an experienced dentist. The undergraduate students had received comprehensive theoretical instruction covering the fundamentals of implant dentistry and had practiced using the implant system in a training setting, specifically, in a typical mandible.

### Fully guided template fabrication

CBCT (Morita Europe GmbH, Dietzenbach, Germany) with a voxel size of 0.125 mm^3^, voltage of 103 kV, tube current set at 6.0 mA, exposure time lasting 9.4 s, and a field-of-view of 100 × 40 mm for the upper jaw and 100 × 50 mm for the lower jaw was performed. The acquired data were transformed into the Digital Imaging and Communications in Medicine (DICOM) format and imported into implant planning software (Organical Dental Implant, Organical CAD/CAM GmbH, Berlin, Germany; coDiagnostiX, Dental Wings GmbH, Chemnitz, Germany and SMOP, Swissmeda AG, Baar, Switzerland).

In addition, an intraoral scan was performed (TRIOS 3, 3Shape Global, Copenhagen, Denmark). The acquired data were transformed into the standard tessellation language (STL) format and imported into the implant planning software. The DICOM data and STL surfaces were matched to plan the virtual implant position. Implant planning considers the local bone volume and the design of future prosthodontic restorations.

Matching was performed by segmenting the radiographic data into only visible hard tissues. Metallic artifacts were removed by segmenting the CBCT data until the bone and teeth were clearly visible, ensuring that superimposition of the CBCT and intraoral scans was as precise as possible. Subsequently, STL data from the intraoral scan were matched to the segmented CBCT image using at least three common surface areas by marking the corresponding dots in both datasets. These dots were selected in a nonlinear manner to achieve three-dimensional superimposition and were placed on easily detectable landmarks, preferably on the teeth, such as fissures and cusp tips. Through the “copy alignment button”, both sets of data were brought into the same coordinate system and geometric position. After alignment, the virtual patient was set up with the important information required to plan an adequate prosthodontic restoration and implant position. First, a tooth corresponding to the missing tooth was selected from the library and placed in a gap. This data served as digital wax-ups. The size, shape, and position of the tooth was modified until satisfactory results were achieved. Therefore, a suitable implant was chosen to be set within the preset prosthetic position and available bone. The safe distances to critical anatomic structures were carefully considered (≥ 2 mm safety distance between planned implant and neighbor teeth, inferior alveolar nerve and floor of the maxillary sinus) [22, 29].

**Fig. 2:**
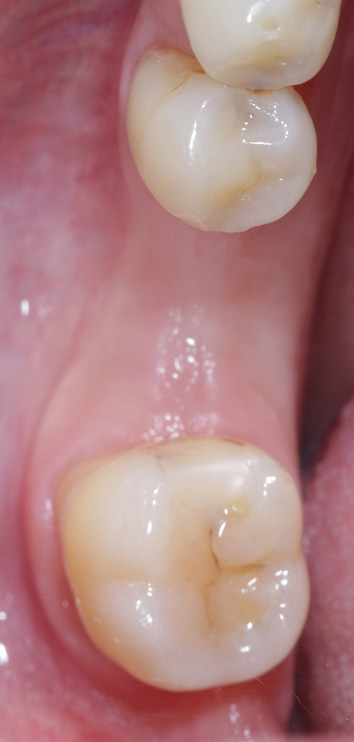
Representative patient with a single tooth gap of region 36

**Fig. 3:**
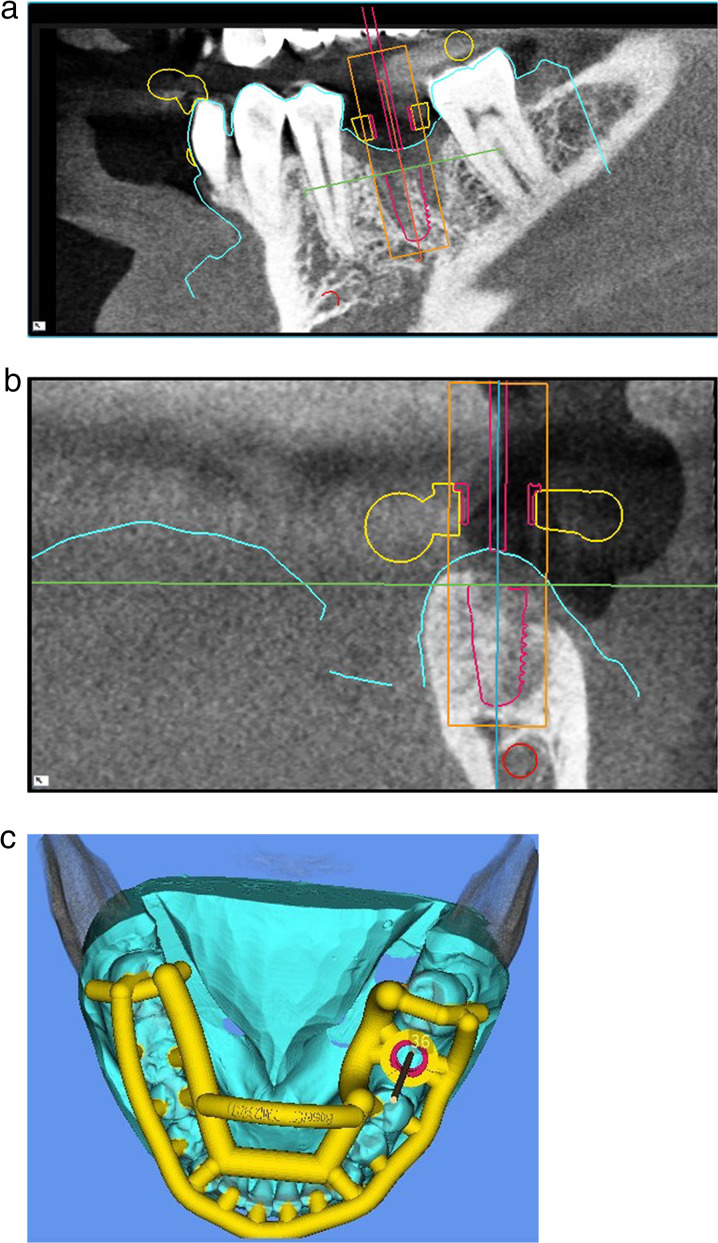
**a**–**c** Virtual implant planning (SMOP, Swissmeda AG, Baar, Switzerland) in region 36. The blue outlines represent the STL data of the intraoral scan which was matched over the CBCT. **c** Shows the design of the surgical template

**Fig. 4:**
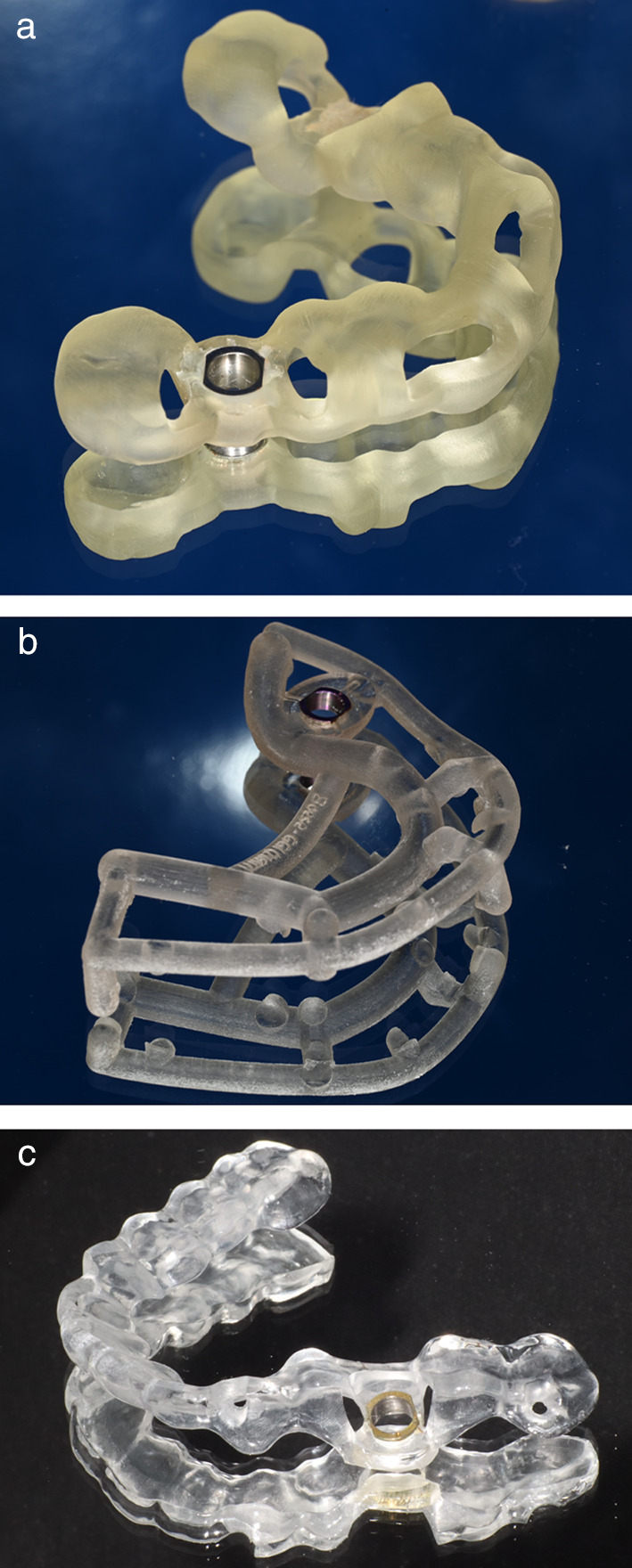
** a**–**c** Surgical guides used in the study. **a** Template for insertion of Straumann implants with 5-mm T-Sleeves (coDiagnostiX). **b** Template for insertion of Camlog implants with 3 mm sleeves (SMOP). **c** Template for insertion of CAMLOG implants with 3 mm sleeves (Organical)

After finishing the implant planning, drill guides were produced by 3D printing (Form 2 and 3B printers, Formlabs GmbH, Berlin; T-Sleeves for the fully guided protocol with the following specifications: Straumann 5 mm and CAMLOG 3 mm; Figs. 2, 3, 4c).

### Surgical protocol

**Fig. 5:**
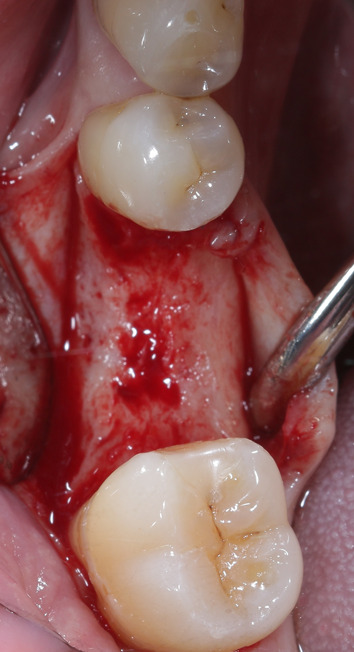
After soft tissue preparation, a sufficient amount of bone volume was presented

**Fig. 6:**
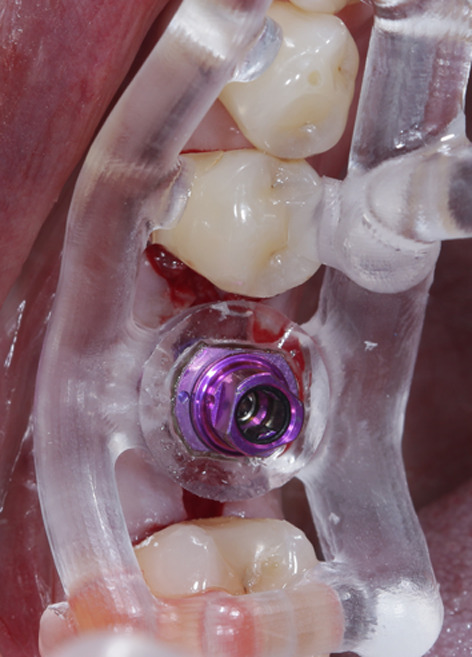
Drilling of the implant bed fully guided through the template

**Fig. 7:**
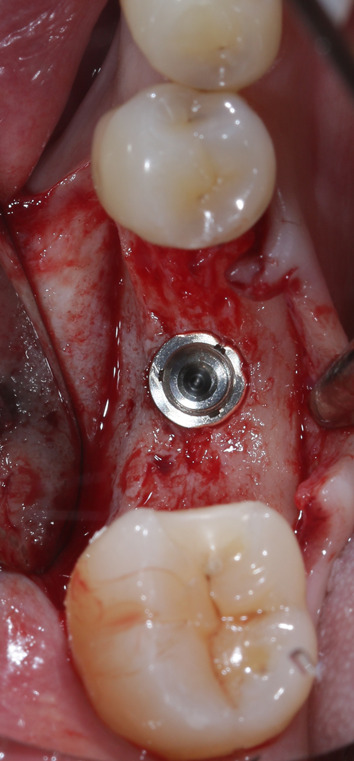
Implant in situ. Following the check for primary stability, an intraoperative scan was performed to determine the real implant position

**Fig. 8:**
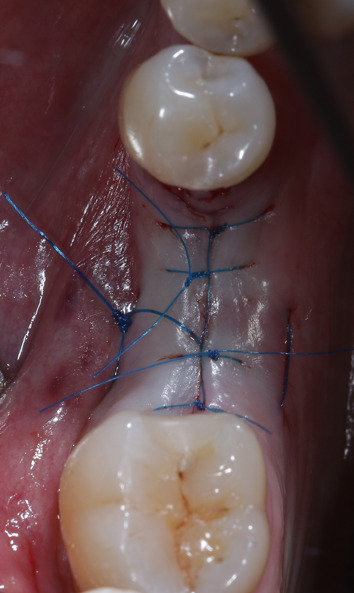
Suturing of the wound. All implants were left to heal subgingivally for 3 months

All surgeries within this study were performed under local anesthesia. Prior to surgery, all patients were instructed to rinse their oral cavity with 0.2% chlorhexidine mouthwash for one minute. After anesthesia, a full-flap approach was used to ensure a sufficient overview (Fig. 5). The surgical template was placed, and its accuracy was thoroughly verified both by tactile assessment and visual assessment via viewing windows in the vestibular part of the template. The recommended drilling protocol was applied to the implant bed prepared for implant reception by rinsing with saline solution. The implants (Straumann Bone Level Tapered, Straumann Holding AG, Basel, Switzerland, and CAMLOG Screw-Line Promote Plus, CAMLOG Biotechnologies AG, Basel, Switzerland) were placed through the drill guide with mounts specifically designed for this purpose. The insertion depth was shown in the surgical protocol and visually followed through checkmarks on the mount. The mount and drill guide were removed from the operating field. Subsequently, a system-specific scan body was screwed onto the implant to determine the postoperative position of the virtually planned implant using an intraoral scanner (TRIOS 3, 3shape, Copenhagen, Denmark). Finally, the scan body was removed, a corresponding closure cap was placed, and the wound was sutured using nonresorbable sutures (Figs. 6, 7, 8). The sutures were removed within eight days. Patients were instructed to rinse their mouth with 0.2% chlorhexidine mouthwash twice a day for the following two weeks, consume soft foods, avoid alcohol and nicotine, and avoid trauma to the specific area through abundant oral hygiene.

### Prosthodontic restoration

**Fig. 9:**
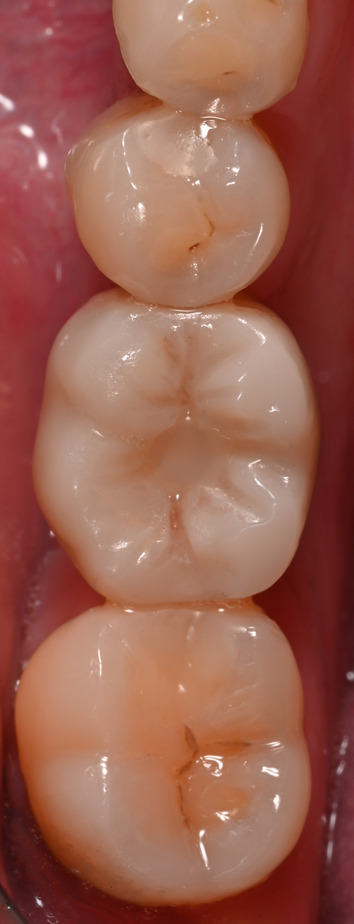
The final restoration, consisting of a screw-retained lithium disilicate crown luted on a titanium base, was delivered

All implants were left to heal subgingivally for at least three months and then uncovered using a drill guide to localize the implant position. An additional intraoral scan was performed to obtain a digital impression of the final prosthodontic restoration. All patients received screw-retained monolithic lithium disilicate crowns on titanium-based abutments (Fig. 9).

### Accuracy evaluation

**Fig. 10:**
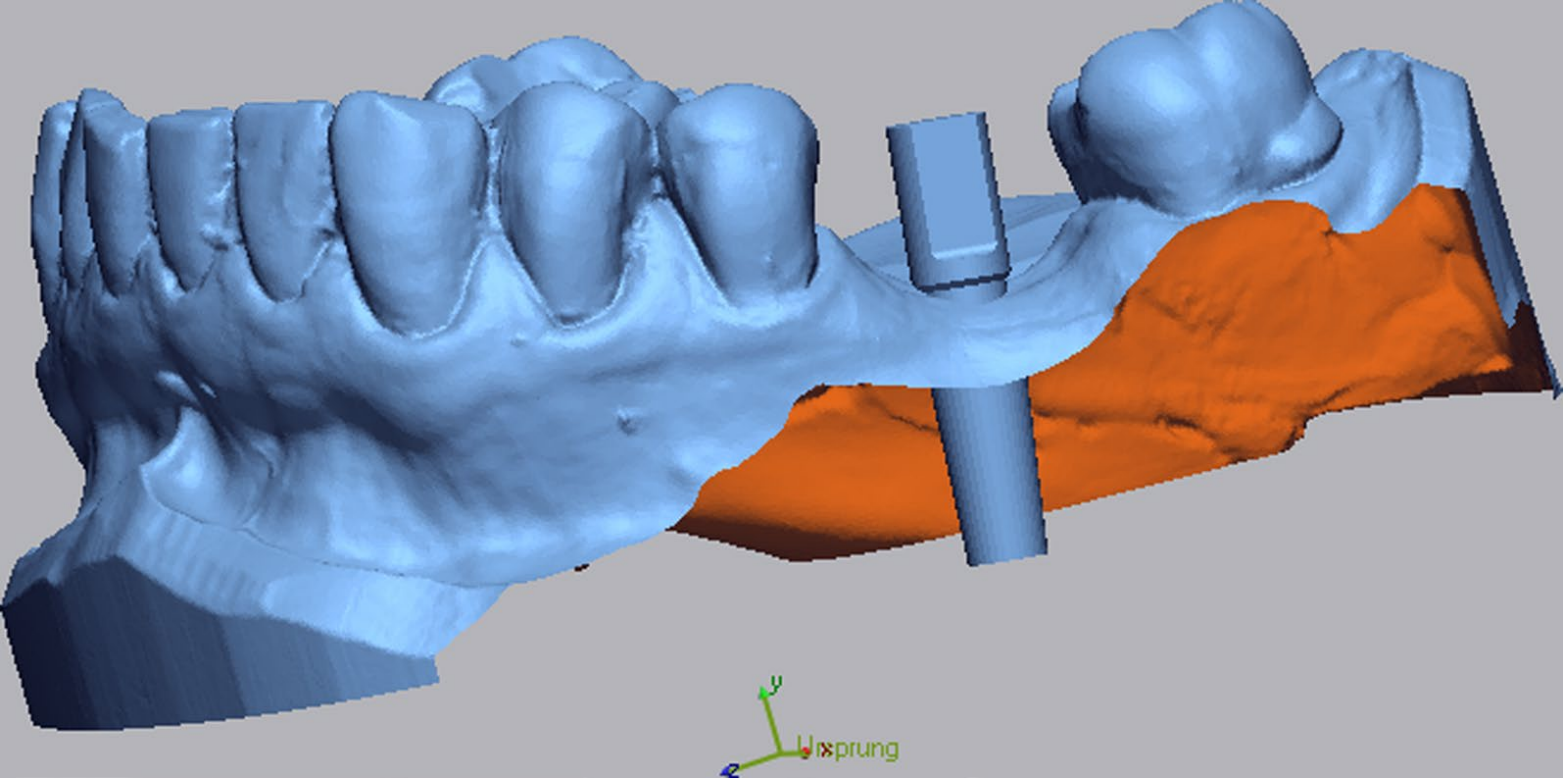
Preparation of the models for measurement: the virtually planned implant

**Fig. 11:**
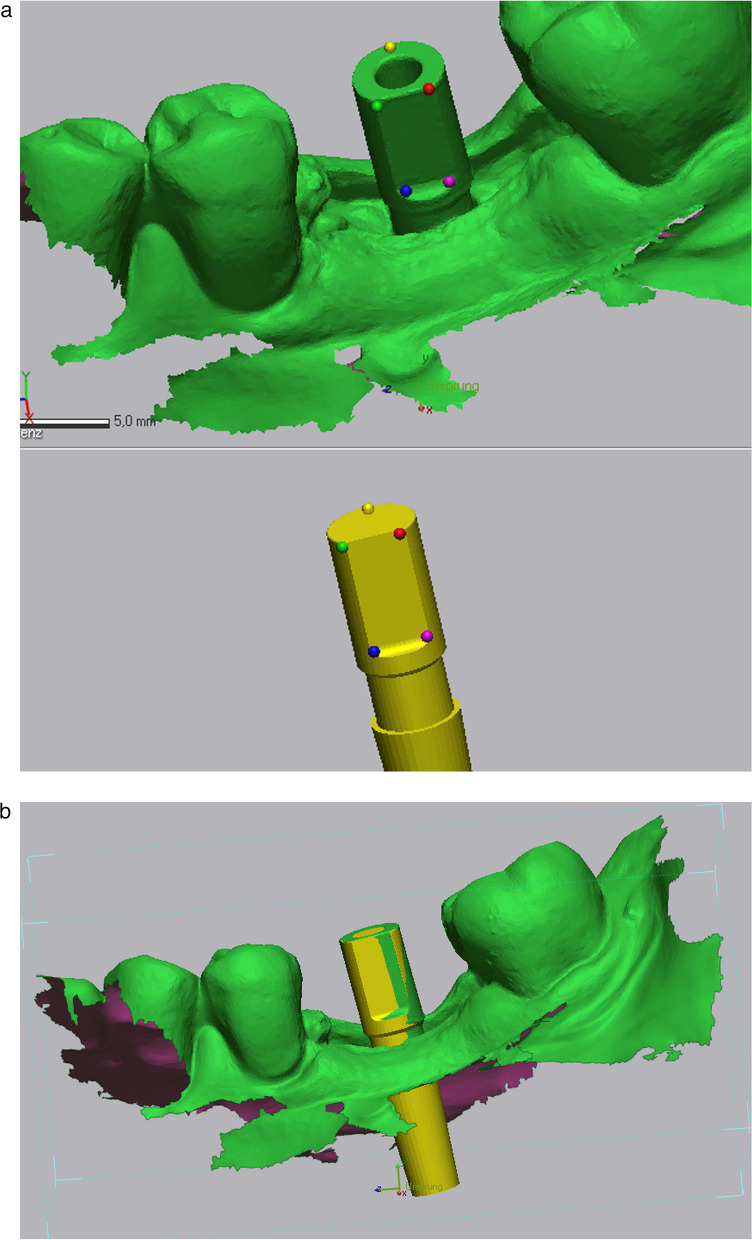
**a**, **b** Preparation of the model illustrating the real implant position: the preplanned implant itself is matched via the scanbody onto the intraoperative scan

**Fig. 12:**
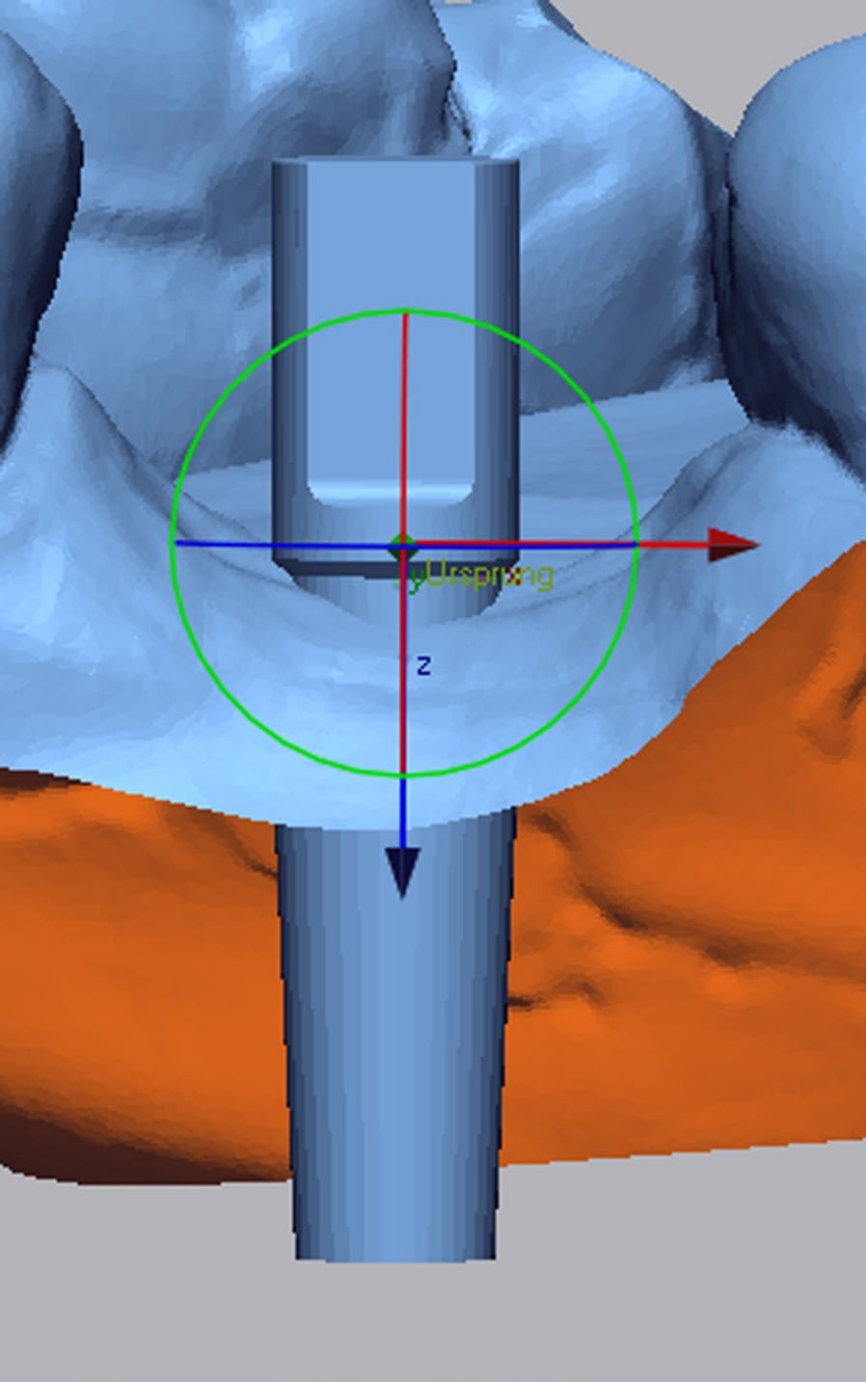
Positioning of the implant within a determined coordinate system for accurate measurements. *X*-axis: mesio-distal direction, *Y*-axis: vestibulo-oral direction, *Z*-axis: coronal–apical direction

**Fig. 13:**
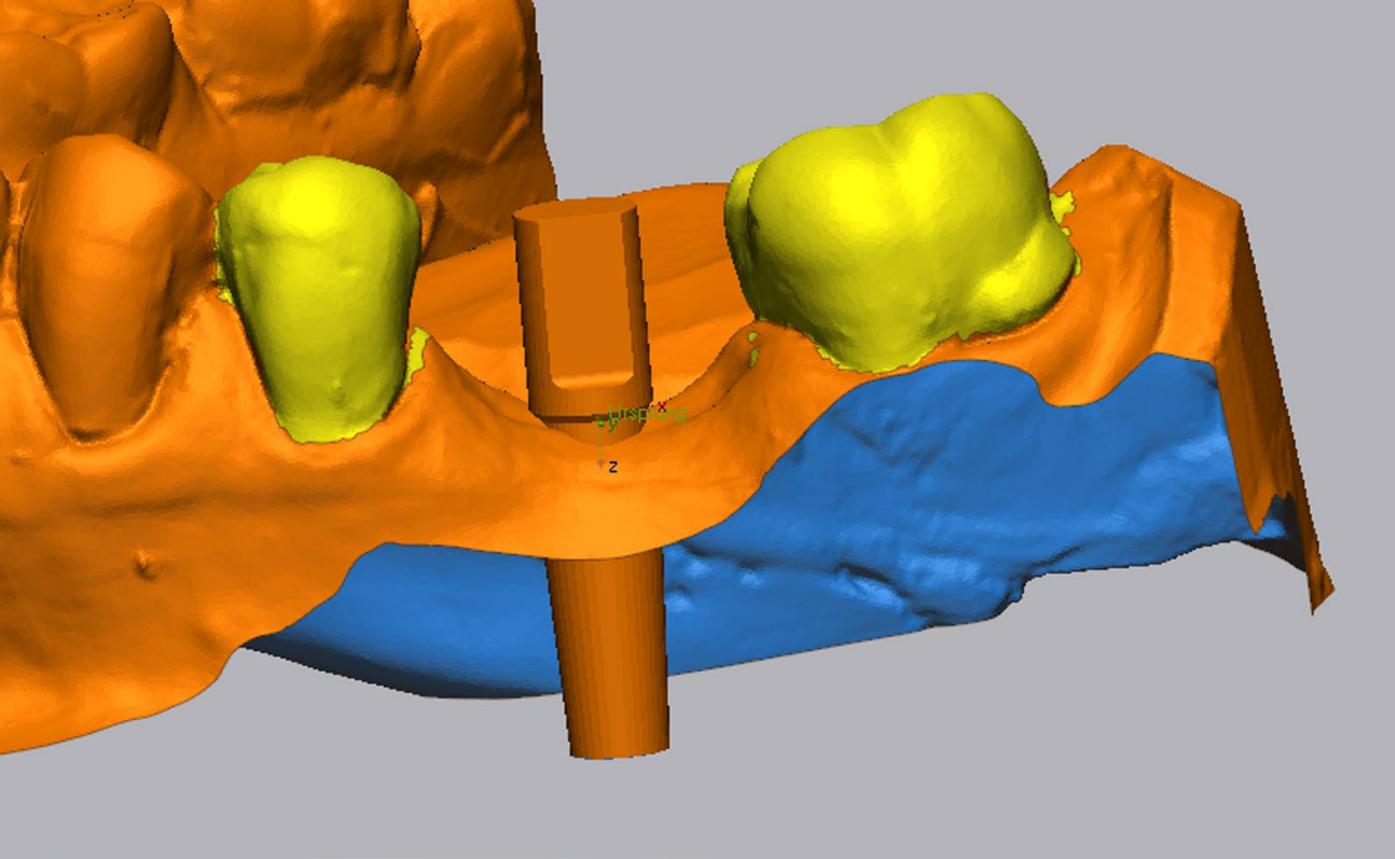
Matching of the real and virtual implant for discrepancy measurements. The implants are matched via the adjacent teeth following best fit alignment

**Fig. 14:**
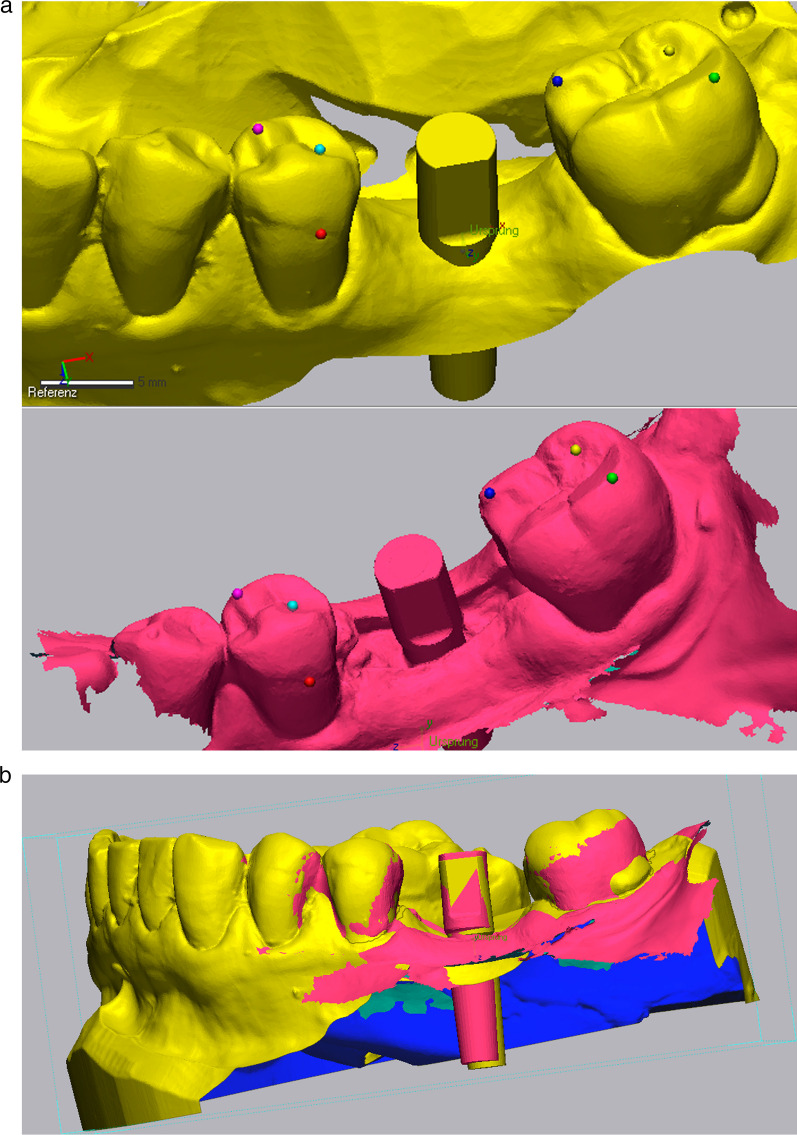
**a**, **b** Matching of the real and virtual implant for discrepancy measurements. The implants are matched via the adjacent teeth following best fit alignment

**Fig. 15:**
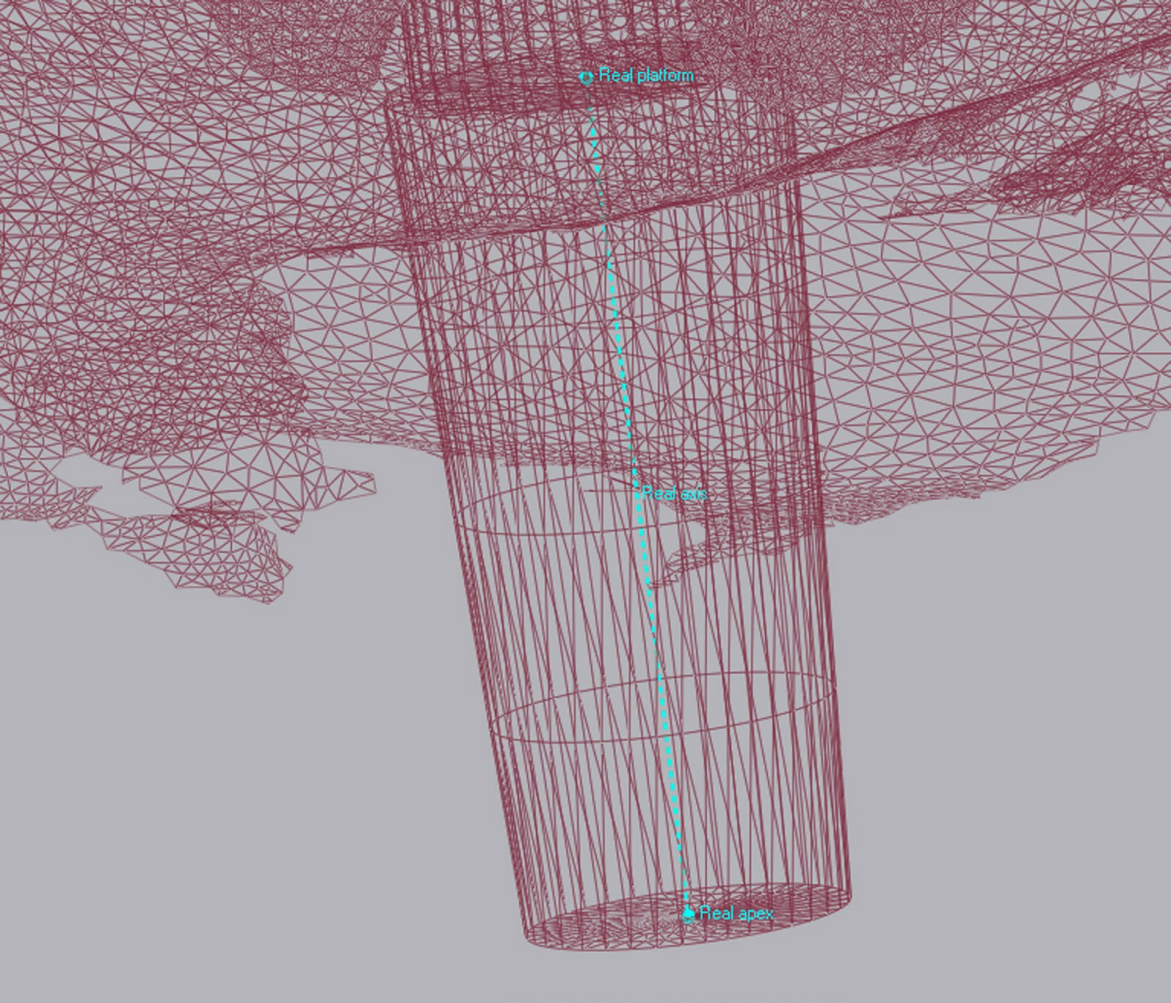
Points were positioned at the centers of the apex and platform of both the virtual and real implants. By connecting these points, the implant axes were determined

**Fig. 16:**
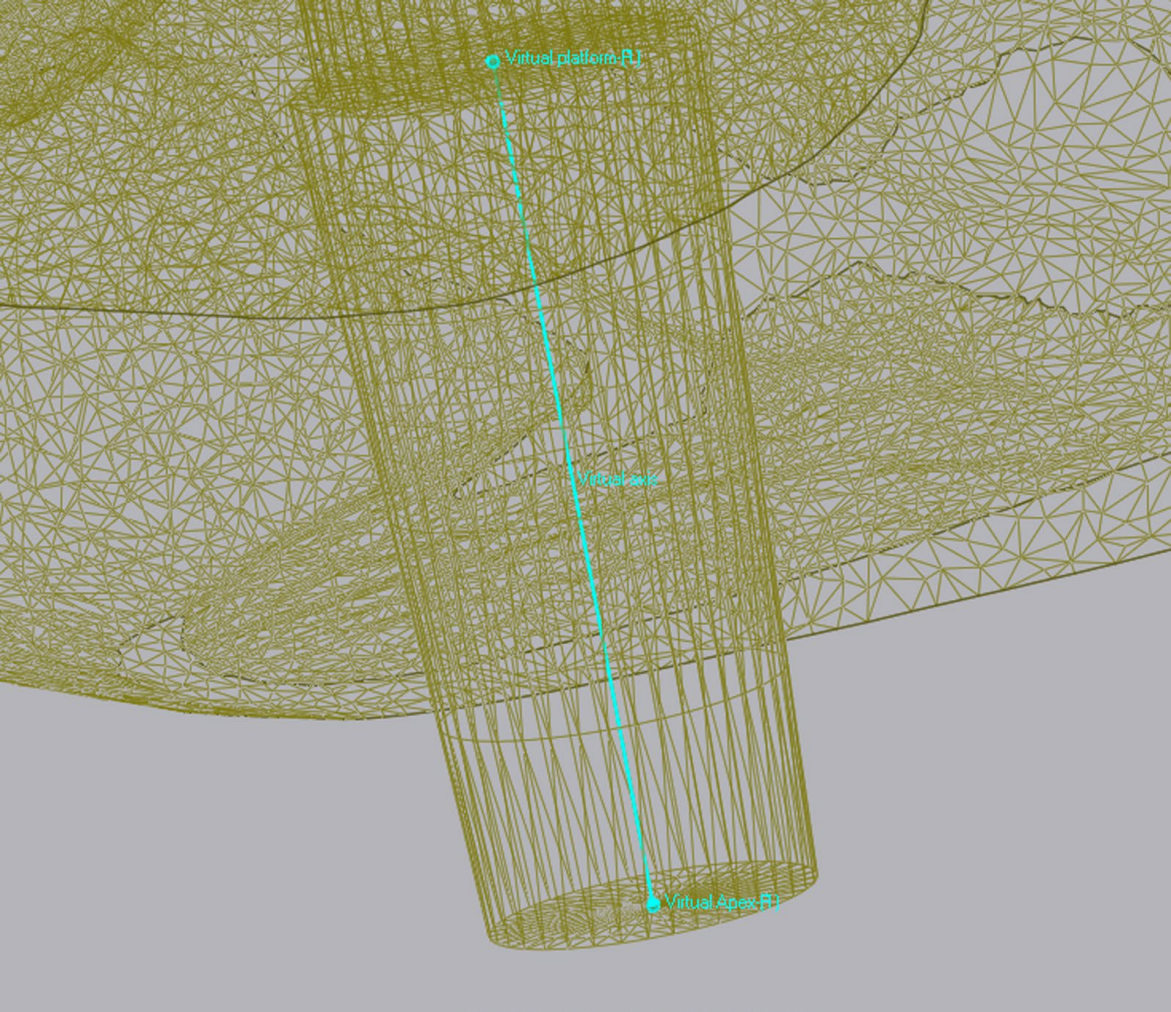
Points were positioned at the centers of the apex and platform of both the virtual and real implants. By connecting these points, the implant axes were determined

**Fig. 17:**
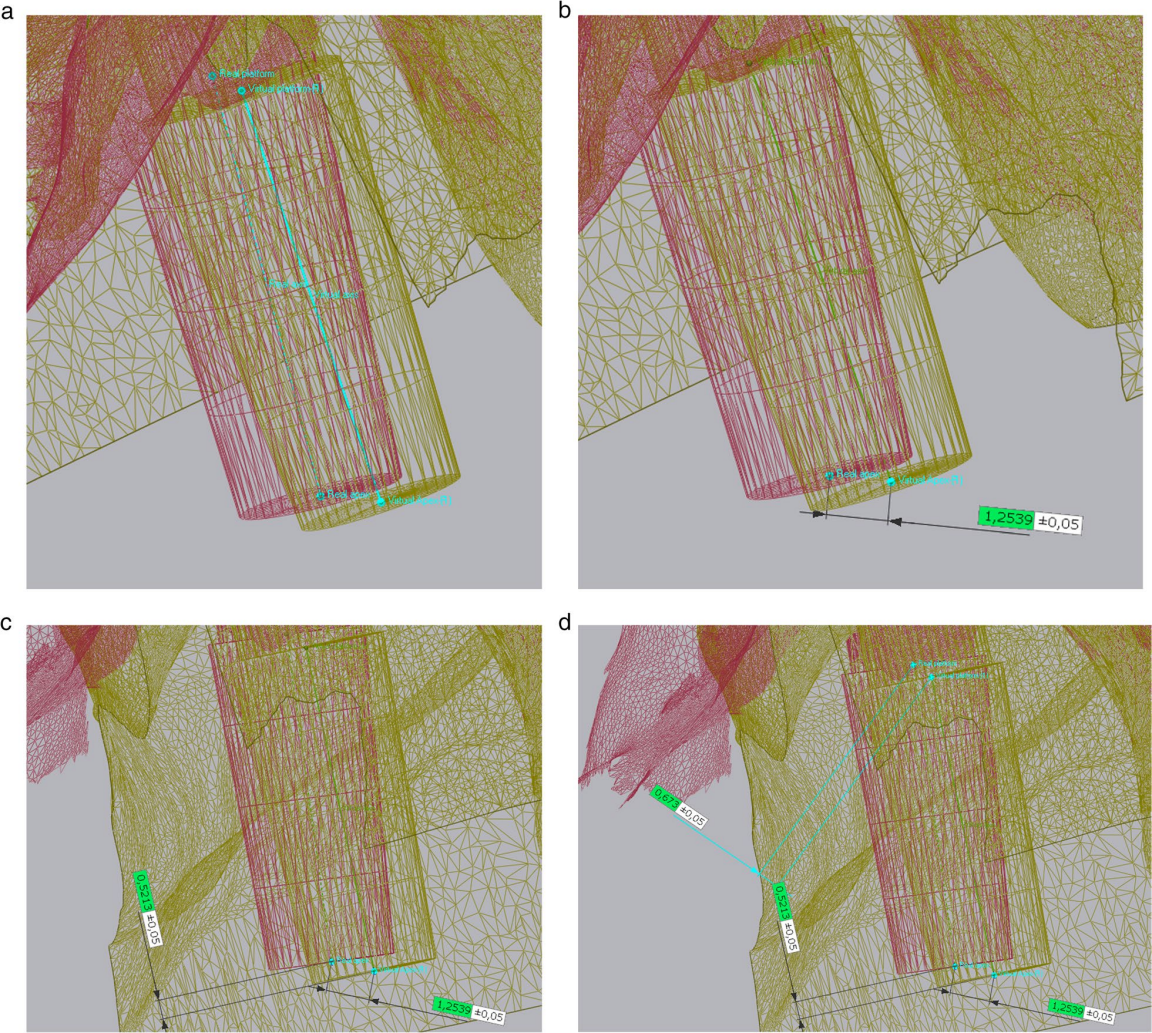
** a**–**d** The measurements were conducted in the following order: **a** closest discrepancy of the apices, **b** vertical discrepancy measured perpendicularly, **c** closest discrepancy of the platforms, and **d** angular deviation of implant axes

**Fig. 18:**
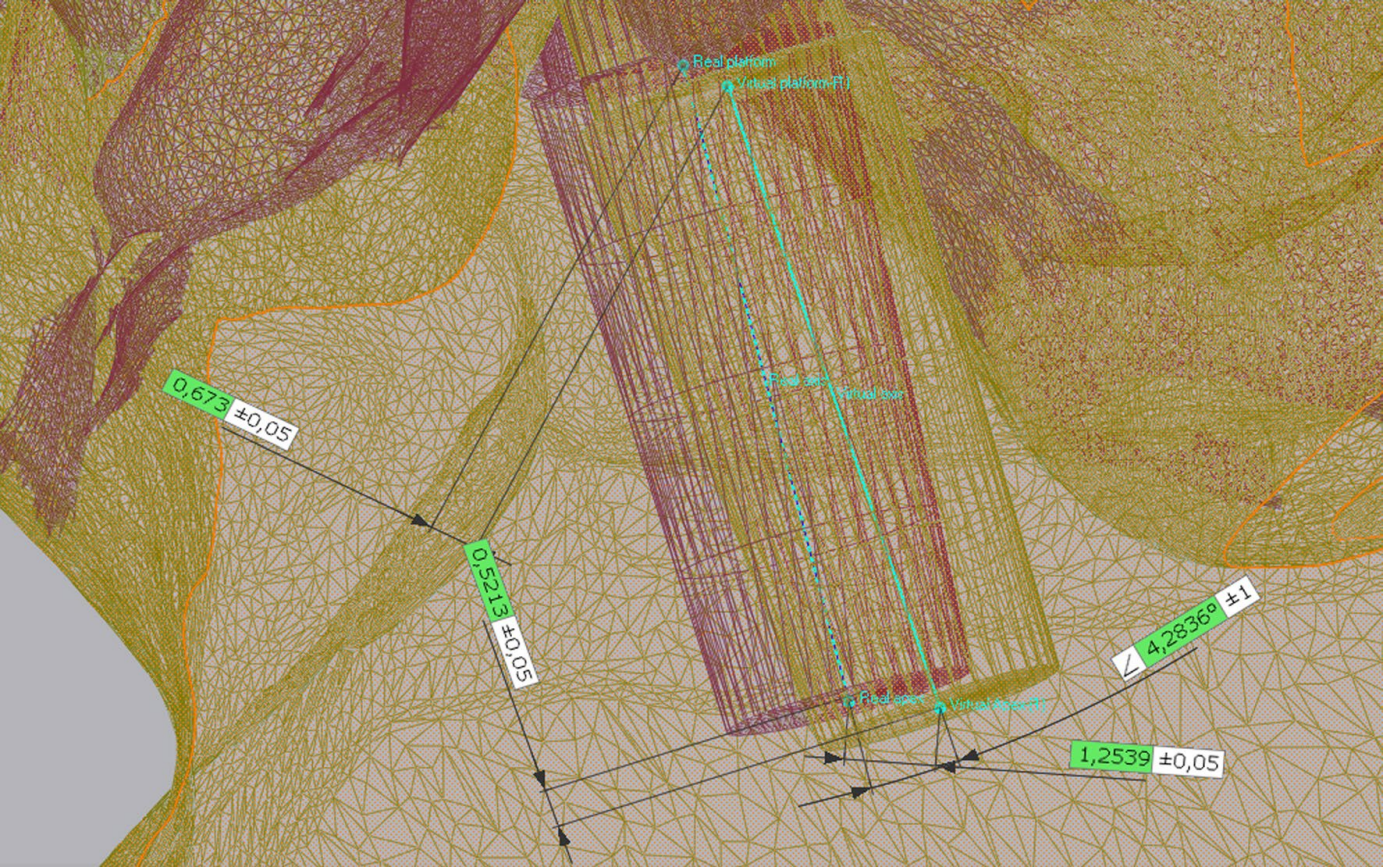
Final measurements at a glance

**Fig. 19:**
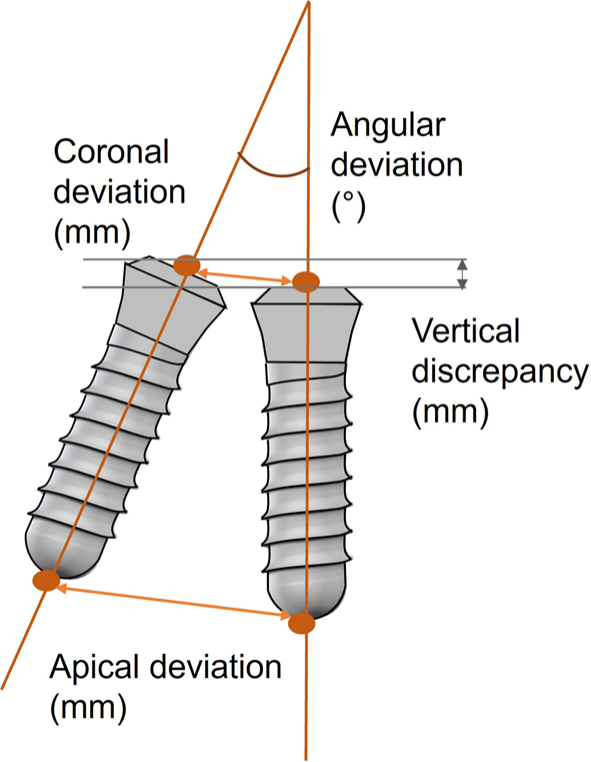
Schematic representation of the evaluation of coronal, apical, and angular deviations, as well as the vertical discrepancy according to Wang et al. [30]

All data collected during implant planning and postoperative scans were processed by a calibrated examiner (E.A., dentist and 3D data specialist), who conducted the entire measurement process using Geomagic Control X 2023.1 (3D Systems Inc., Rock Hill, South Carolina, USA). The planning data included the intraoral scan from the initial stage, the implant, and the scan body complex representing the virtual implant position. From these data, a set of implants and scan bodies was formed, exported as a whole, and matched to the scan body of the postoperative scan, following the same matching principle (Figs. 10, 11, 12). This allowed the creation of a model that represented the actual implant position. Next, the models of the virtual and real implant positions were matched for measurements. This was conducted by matching the two adjacent teeth to a gap of at least three dots in the remarkable landmarks (Figs. 13, 14a, b). In addition, automatic best-fit alignment was implemented over the same teeth to provide the most accurate alignment (Figs. 15, 16). Four dots were placed in the apical and coronal centers of the virtual and real implants. Finally, the distances between the apical and coronal points between the virtual and real implant positions were measured in millimeters, as well as the angular deviation of both implants in degrees (Fig. 17a–c). In addition, the vertical discrepancies were determined. In contrast to the first measurement which displayed the closest distance between the set points, this measurement was conducted perpendicularly using only the z-axis as the measuring element (Fig. 17d). Thus, the parameters for determining the mismatch between the planned and inserted implant positions were the apical deviation (mm), coronal deviation (mm), angular deviation (°), and vertical discrepancy (mm) (Figs. 18, 19).

### Statistical analysis

Statistical analyses were performed using SPSS Statistics, version 28 (IBM Corporation, Armonk, New York, USA). After descriptive data analysis, the Kolmogorov–Smirnov and Shapiro–Wilk tests were used to evaluate whether the values were normally distributed. The measured data of the two groups (angulation, mismatch at the implant base and implant tip, and vertical discrepancy) were compared using Mann–Whitney *U*-tests. The level of statistical significance was set at *α* = 0.05.

## Results

### Operators, patient recruitment and descriptive analysis

**Fig. 20:**
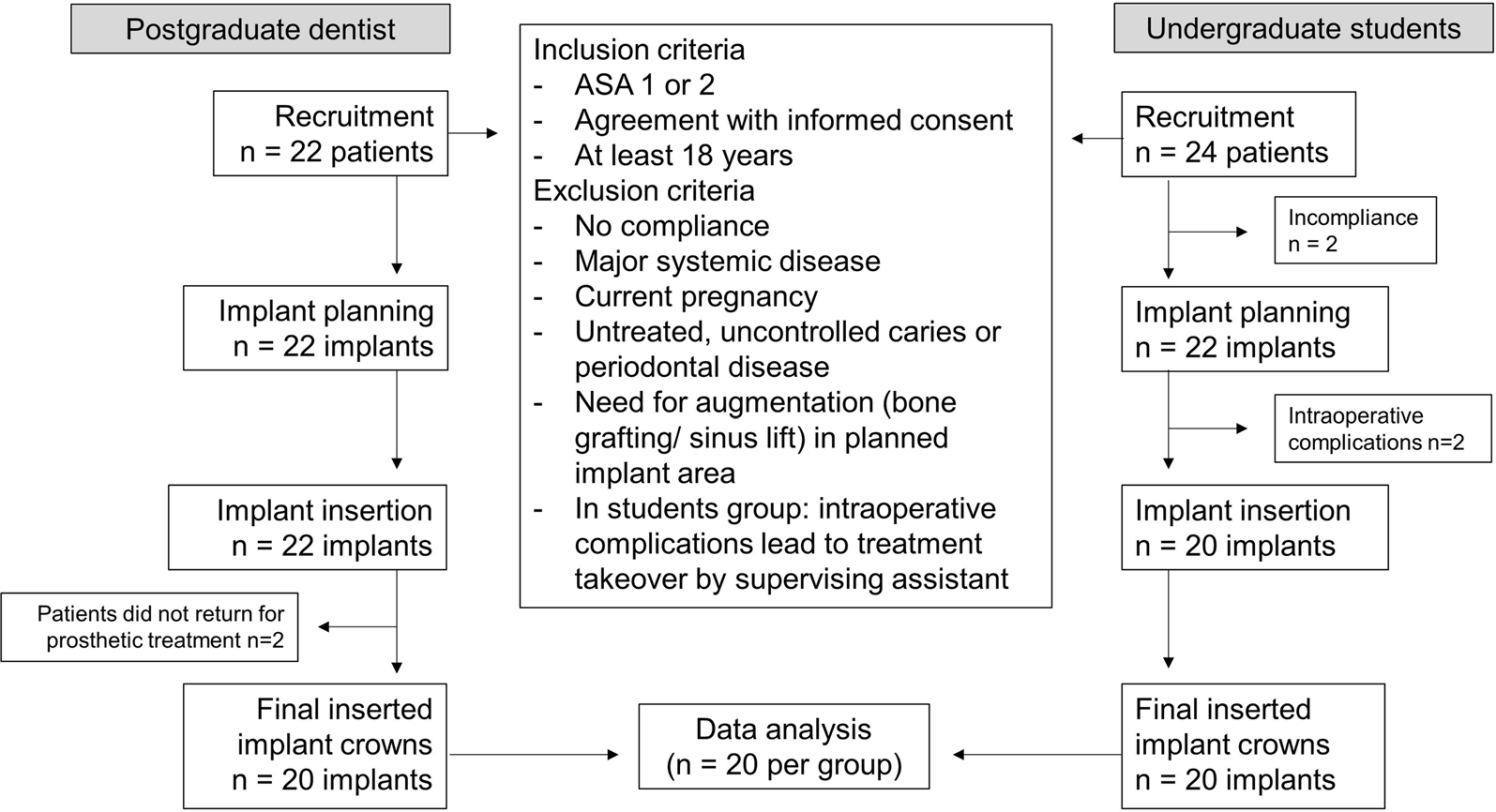
The study process including the inclusion and exclusion criteria, as well as number of patients and inserted implants

Forty-four patients with indications for 46 implants were enrolled in the study. Six patients were excluded before or during the study according to the predefined exclusion criteria (Fig. 20). Of the 38 patients finally included in the study, 20 patients were treated by the postgraduate dentists (*n* = 5) at the Department of Prosthodontics, Geriatric Dentistry, and Craniomandibular Disorders, Charité University Medicine Berlin, and 18 patients were treated by undergraduate students (*n* = 18) in the Department of Prosthodontics, Martin-Luther-University Halle-Wittenberg (Table 1).

**Table 1:** Characteristics of the participants and patients

Group	Participants (dentists/students) characteristics		Patients characteristics	
	Number (male/female)	Age (mean ± SD, years)	Number (male/female)	Age (mean ± SD, years)
Postgraduate dentists	*n* = 5 (2/3)	31.5 ± 1.5	*n* = 20 (11/9)	51.9 ± 14.4
Undergraduate students	*n* = 18 (5/13)	26.1 ± 4.3	*n* = 18 (11/7)	40.4 ± 12.9

Twenty implants were inserted by postgraduate dentists (CAMLOG, *n* = 9; Straumann, *n* = 11) and undergraduate students (CAMLOG, *n* = 20). The implant characteristics for each group are listed in Table 2.

**Table 2:** Characteristics of the implants

Group	Implant system	Implant diameter (mm)	Implant length (mm)				Jaw and gap				Implant site (FDI)
							Upper jaw		Lower jaw		
			8	9	10	11	Inter-dental gap	Free end	Inter-dental gap	Free end	
			*n*	*n*	*n*	*n*	*n*	*n*	*n*	*n*	
Postgraduate dentist	Straumann	3.3	0	0	3	0	2	0	1	0	14, 24, 46
		4.1	2	0	4	0	1	0	5	0	27, 35, 3 × 36, 45
		4.8	0	0	2	0	0	0	2	0	2 × 36
	CAMLOG	4.3	0	5	0	4	1	0	7	1	16, 3 × 36,2 × 37,3 × 46
Undergraduate students	CAMLOG	4.3	0	4	0	7	1	0	7	3	24, 6 × 36, 45, 3 × 46
		3.8	0	7	0	2	5	0	2	2	2 × 15, 24, 26, 2 × 35, 36, 46

*FDI* Fédération Dentaire Internationale

No complications or implant losses were reported during the follow-up period. Consequently, the current survival rate stands at 100%. Table 3 presents the specific time intervals between implant insertion, implant exposure, and prosthetic insertion for both groups.

**Table 3:** Time intervals between implant insertion, uncovery, and prosthetics restoration depending on the operator

Group	Healing time before implant exposure	Time between insertion of implant and prosthetics	Implant survival after implant insertion	Implant survival after insertion of prosthetics
	Days (mean ± SD)			
Postgraduate dentists	88 ± 110.4	243.5 ± 138.4	834.5 ± 172.5	598.33 ± 195
Undergraduate students	142.9 ± 66.9	218.3 ± 103.9	1859.3 ± 262.7	1641 ± 302.1

Table 4 summarizes the results for coronal, apical, and angular deviations, as well as vertical discrepancies, to enable comparisons of planned and placed implant positions based on the operator, gap situation, jaw assignment, and implant length. The Kolmogorov–Smirnov and Shapiro–Wilk tests indicated that the four predefined implant accuracy parameters did not follow a normal distribution. Thus, the Mann–Whitney *U*-Test was used for pairwise comparisons between different groups. Regardless of the variable under investigation, no significant differences were found between the respective groups (Table 4).

**Table 4:** Mismatch between planned and placed implant position regarding apical, coronal and angulation deviation as well as vertical discrepancy (mean, standard deviation [SD], minimum [min], maximum [max]) according to the operator, gap situation, jaw assignment, and implant length

Factor potentially influencing implant accuracy	Group affiliation (number of implants)	Apical deviation				Coronal deviation				Angulation deviation				Vertical discrepancy			
		Mean ± SD (mm)	Min (mm)	Max (mm)	*p*-value	Mean ± SD (mm)	Min (mm)	Max (mm)	*p*-value	Mean ± SD (°)	Min (°)	Max (°)	*p*-value	Mean ± SD (mm)	Min (mm)	Max (mm)	*p*-value
Operator	Postgraduate dentists (*n* = 20)	0.97 ± 0.35	0.25	1.70	0.245	0.63 ± 0.25	0.09	1.01	0.745	2.92 ± 1.16	1.08	5.13	0.185	0.47 ± 0.24	0.05	0.90	0.433
	Undergraduate students (*n* = 20)	1.17 ± 0.58	0.18	2.42		0.80 ± 0.56	0.14	2.04		3.75 ± 1.86	1.54	8.56		0.56 ± 0.60	0.02	2.01	
Gap situation	Inter-dental gap (*n* = 34)	1.01 ± 0.43	0.19	2.12	0.130	0.66 ± 0.40	0.09	2.01	0.069	3.29 ± 1.61	1.08	8.56	0.622	0.46 ± 0.42	0.02	2.01	0.096
	distal extension (*n* = 6)	1.41 ± 0.63	0.75	2.42		1.03 ± 0.55	0.52	2.04		3.55 ± 1.60	1.73	5.34		0.81 ± 0.56	0.34	1.85	
Jaw	Upper (*n* = 10)	1.11 ± 0.48	0.66	2.12	0.827	0.86 ± 0.50	0.26	2.01	0.274	3.51 ± 1.73	1.74	6.81	1.00	0.59 ± 0.58	0.04	2.01	0.794
	Lower (*n* = 30)	1.05 ± 0.49	0.19	2.42		0.67 ± 0.41	0.09	2.04		3.27 ± 1.57	1.08	8.56		0.49 ± 0.41	0.02	1.85	
Implant length	8–9 mm (*n* = 18)	1.15 ± 0.48	0.42	2.12	0.328	0.81 ± 0.45	0.19	2.01	0.174	3.17 ± 1.33	1.54	6.54	0.587	0.54 ± 0.52	0.04	2.01	0.957
	10–11 mm (*n* = 22)	1.01 ± 0.49	0.19	2.42		0.64 ± 0.42	0.09	2.04		3.47 ± 1.79	1.08	8.56		0.49 ± 0.40	0.02	1.85	

As the postgraduate dentists used two different implant systems (CAMLOG and Straumann), differences in implant accuracy between the two systems were also analyzed in this group. However, no statistically significant differences were found in apical (*p* = 0.245), coronal (*p* = 0.745), or angular (*p* = 0.185) deviations or vertical discrepancies (*p* = 0.433).

In two cases within the student group, insufficient primary stability occurred, leading to the intervention of the supervising experienced dentist, who assumed control of the treatment. After additional freehand expansion of the implant bed, an implant with a wider diameter was inserted without use of the surgical template. These two implants were excluded from the general analysis; however, the differences between the accuracies of fully guided and partially guided implants, without the use of the drill guide, are shown in Table 5. Statistically significant differences were observed in apical and angular deviations.

**Table 5:** Mismatch between planned and placed implant positions regarding apical, coronal, and angulation deviation as well as vertical discrepancy (mean, standard deviation [SD], minimum [min], maximum [max]) according to implant insertion technique

Implant insertion	Apical deviation				Coronal deviation				Angulation deviation				Vertical discrepancy			
	Mean ± SD (mm)	Min (mm)	Max (mm)	*p*-value	Mean ± SD (mm)	Min (mm)	Max (mm)	*p*-value	Mean ± SD (°)	Min (°)	Max (°)	*p*-value	Mean ± SD (mm)	Min (mm)	Max (mm)	*p*-value
Fully guided (*n* = 20)	1.17 ± 0.58	0.18	2.42	0.022	0.80 ± 0.56	0.35	2.04	0.068	3.75 ± 1.87	1.54	8.56	0.022	0.56 ± 0.60	0.02	2.01	0.248
Partially guided (*n* = 2)	3.41 ± 0.06	3.37	3.45		1.81 ± 0.12	1.73	1.89		9.96 ± 1.14	9.16	10.77		1.31 ± 0,61	1.16	1.47	

## Discussion

The analysis of the deviations between planned and placed implant positions showed that there were deviations both in the group of postgraduate dentists and in the group of undergraduate students. However, no significant differences were found between the two groups, thus, the null hypothesis was accepted.

Apical deviations of 0.97 ± 0.35 mm, coronal deviations of 0.63 ± 0.25 mm, angulation deviations of 2.92 ± 1.16°, and a vertical deviation of 0.47 ± 0.24 mm were found in the postgraduate dentists’ group. In the undergraduate students’ group, apical deviations of 1.17 ± 0.58 mm, coronal deviations of 0.80 ± 0.56 mm, angulation deviations of 3.75 ± 1.86°, and vertical discrepancies of 0.56 ± 0.60 mm were determined. Thus, the range of these discrepancies between the planned and placed implant positions is in line with the results in the literature. Rungcharassaeng et al. and Cassetta and Bellardini presented comparable deviations in inexperienced practitioners (coronal deviations of 0.64 ± 0.21 mm, apical deviations of 1.22 ± 0.63 mm, vertical deviations of − 0.51 ± 0.21 mm and angulation deviations of 3.21 ± 1.99° vs. coronal deviations of 0.75 ± 0.18 mm, apical deviations of 1.02 ± 0.44 mm, and angulation deviations of 3.07 ± 2.70°, respectively) [31, 32]. Interestingly, both studies also found deviations between planned and placed implant positions in experienced practitioners, although they did not significantly differ from those of inexperienced practitioners (Rungcharassaeng et al.: coronal 0.47 ± 0.15, apical 1.32 ± 0.25, vertical − 0.26 ± 0.23, angulation 4.11 ± 0.76°; Cassetta and Bellardini: coronal 0.60 ± 0.25 mm, apical 0.67 ± 0.34 mm, angulation 3.21 ± 1.57°). This phenomenon can be explained by the fact that inherent errors in computer-guided implant surgery can lead to imprecise implant placement, regardless of the experience of the operator [33, 34]. In addition to intraoperative errors, which could be due to the lack of expertise of the surgeon, incorrect matching of CBCT and scan data, errors in the preparation of the surgical template, and fixation of the guiding sleeve [31, 35]. In a systematic review, Schneider et al. reported mean coronal, apical, and vertical deviations of approximately 1.07 mm, 1.63 mm, and 0.43 mm, respectively, and a mean angular deviation of 5.26 degrees [33].

Interestingly, in the present study and other previous studies, coronal deviations were found to be smaller than apical deviations. D'Haese et al. explained this by pointing out that minimal coronal drilling defects can result in axial deviation in bone depth, leading to larger apical deviations [36], especially for longer implants. In the current study, the analysis of the influence of implant length did not reveal relevant differences between shorter and longer implants. Nevertheless, the significance of these results should be regarded as very weak. This is attributed to the considerable variation in group sizes for implants with lengths of 8, 9, 10 and 11 mm, which were combined into two groups (8 to 9 mm and 10 to 11 mm) for the statistical analysis.

However, the question of whether the deviations found are clinically relevant remains unanswered. Di Giacomo et al. divided the apical and coronal deviations into slight deviations (≤ 1 mm), moderate deviations (> 1 to ≤ 2 mm), and relevant deviations (≥ 2 mm) [37]. Considering this classification, the mean deviations found in this study should be considered as slight to moderate in both groups. However, this did not apply to the two implants that had to be inserted without the use of a surgical template by the supervising assistant because of the lack of primary stability after fully guided preparation of the implant bed. In these cases, the greatest deviation between planned and placed implant positions were measured.

The lack of primary stability has been highlighted in the literature as one of the most common problems in fully guided implant surgery [25, 37, 38]. In the present study, the deviation from the surgical protocol led to significantly higher deviations than in implants placed according to the fully guided protocol (apical 3.41 ± 0.06, coronal 1.81 ± 0.12, vertical 1.31 ± 0.61, and angulation deviations of 9.96 ± 1.14°). Due to the minimal sample size (*n* = 2), these findings have to be critically evaluated. However, a meta-analysis by Putra et al. showed that freehand insertion after pilot drilling led to greater deviations than fully guided implantation [23]. Even under ideal in vitro conditions, higher deviations that were comparable to those in the present study were observed with freehand insertion [39]. The apical deviations found in this study support the recommendations of Mistry et al., who proposed that, in the context of freehand implantations, safety distances of 3 mm should be taken into account to prevent severe consequential damage [22].

The literature has discussed the significance of the surgeon’s experience as instrumental in achieving precision in implantation [1, 9]. The present data indicate that postgraduate dentists tended to achieve smaller deviations from the planned implant position than students, but this difference was not found to be statistically significant or clinically relevant. However, Choi et al. and Cushen et al. demonstrated that the precision of implant placement improved with the increasing surgical experience of the surgeon [20, 40]. In the present study, postgraduate dentists already had more surgical experience than the undergraduate students based on at least two years of clinical experience. It is possible that the training resulted in the dentists achieving an overall higher level of accuracy than the students. However, it is important to note that there was still no significant and clinically relevant difference, which is an additional indication of the safety of the fully guided method.

Regarding the implant survival rate, no differences were found between the two groups as no implant loss or postoperative complications were observed during the observation period. However, it is important to note that the significance of this result is limited by the relatively short follow-up period of 598.33 ± 195 days (postgraduate dentists) and 1641 ± 302.1 days (undergraduate students), indicating that long-term survival cannot be evaluated in this context (see Table 3). It was also observed that the implant healing time (88 ± 110.4 days for postgraduate dentists vs. 142.9 ± 66.9 days for undergraduate students), as well as the time until integration of the prosthetic restoration (243.5 ± 138.4 days for postgraduate dentists and 218.3 ± 103.9 days for undergraduate students), differed between both groups. This can be attributed to logistic challenges in student training (e.g., semester breaks) as well as the fact that, in the undergraduate students’ group, solely implant-supported fixed prosthodontics were more frequently fabricated, whereas in the postgraduate dentists’ group, prosthetic treatment included more comprehensive prosthetic restorations.

Besides the surgeon’s experience, various other factors that influence the precision of fully guided implant placement have been discussed in the literature. For example, whether the use of a flapless protocol can lead to less deviation in the implant position has been discussed. Although some studies showed better outcomes with the flapless protocol [23, 41], this was not confirmed in other studies [42]. In the present study, a flap was created to improve the surgical overview. The extent to which flapless surgery would have reduced the discrepancies between the planned and inserted implants can only be speculated. However, in the two cases in which freehand implant placement was required due to a lack of primary stability, the flap that had already formed proved to be extremely helpful in retrospect.

In addition, an assessment was conducted to determine whether affiliation to the jaw led to varying deviations between the planned and placed implant positions. In the present study, smaller deviations were observed for implants placed in the mandible than those placed in the maxilla. This tendency was also reported by Ersoy et al. [42]. However, clinical relevance has not yet been reported [23, 42].

The edentulous area has also been discussed as a factor influencing the precision of fully guided procedures [23]. In the present study, implants were placed only in patients with minor tooth loss and the templates were well supported by the available teeth. Similar to the results of the present study, Raico Gallardo et al. found smaller deviations between the planned and placed implant positions in implant placement in interdental gaps than in free-end situations [43]. This is because of the better fixation of the template without the risk of lateral thrust in the interdental gaps. Furthermore, there is a risk of displacement of the surgical template due to differently resilient mucosa, mucosa thickness, and possibly swelling due to local anesthesia in free-end situations [23, 43].

Several other limitations should be considered when evaluating the results of this study. Overall, although our results were comparable with those reported in existing literature, the number of implant cases and participants both in the undergraduate students’ and postgraduate dentists’ groups were comparatively small. Moreover, the postgraduate dentists used two different implant systems and planning software, whereas the undergraduate students used only one. Nevertheless, because the evaluation of implant accuracy depending on the implant system showed no significant difference in the postgraduate dentist group, all implants were included in the analysis. This decision was based on results of the literature that described comparable implant accuracy between different planning and implant systems [29].

The informative value of the study is further limited by the fact that only straightforward cases were selected, based on the SAC classification [44]. The extent to which novice practitioners achieve better results than undergraduate students in more difficult cases (e.g., partially edentulous or edentulous patients) should be the subject of future investigation.

Finally, it must be noted that in two cases within the group of undergraduate students, the operation had to be performed by the supervising assistant. As a result, the final implant bed preparation and implant placement took place without the previously constructed template, which resulted in significantly higher deviations from the planned and achieved implant position. This raises the question of whether dynamic implant placement might not have provided significant advantages in such cases. This technique is supported by a computed-navigation system, in which planned implant position and real-time position of the drill tip could be visualized. Thus, the preoperatively intended implant location as well as implant configuration can be changed in real-time in a controlled way when needed [45, 46]. Future investigations should focus on the potential benefits of dynamic implantation in undergraduate dental education.

## Conclusions

The deviations observed in this study demonstrated smaller, however not statistically significant, discrepancies in planned and placed implant positions in the group of postgraduate dentists compared to the undergraduate students. The deviations observed fell within the range reported in the existing literature, affirming the feasibility of employing the fully guided method in undergraduate training. However, it is advisable to have an experienced supervisor on hand to provide guidance and support as needed. Further investigation into the practical benefits and limitations of employing this method in different clinical scenarios is warranted.

## Data Availability

The datasets used and/or analyzed during the current study are available from the corresponding author on reasonable request.

## Abbreviations

CBCT: Cone beam computer tomography
STL: Standard tessellation language
